# Magnetic mechanism for the biological functioning of hemoglobin

**DOI:** 10.1038/s41598-020-64364-y

**Published:** 2020-05-22

**Authors:** Selma Mayda, Zafer Kandemir, Nejat Bulut, Sadamichi Maekawa

**Affiliations:** 1 Department of Physics, Izmir Institute of Technology, Urla, 35430 Turkey; 2 Department of Materials Science and Engineering, Izmir Institute of Technology, Urla, 35430 Turkey; 3 RIKEN Center for Emergent Matter Science, Wako, 351-0198 Japan; 4 Kavli Institute for Theoretical Sciences, University of Chinese Academy of Sciences, Beijing, 100049 China

**Keywords:** Metalloproteins, Magnetic properties and materials

## Abstract

The role of magnetism in the biological functioning of hemoglobin has been debated since its discovery by Pauling and Coryell in 1936. The hemoglobin molecule contains four heme groups each having a porphyrin layer with a Fe ion at the center. Here, we present combined density-functional theory and quantum Monte Carlo calculations for an effective model of Fe in a heme cluster. In comparison with these calculations, we analyze the experimental data on human adult hemoglobin (HbA) from the magnetic susceptibility, Mössbauer and magnetic circular dichroism (MCD) measurements. In both the deoxygenated (deoxy) and the oxygenated (oxy) cases, we show that local magnetic moments develop in the porphyrin layer with antiferromagnetic coupling to the Fe moment. Our calculations reproduce the magnetic susceptibility measurements on deoxy and oxy-HbA. For deoxy-HbA, we show that the anomalous MCD signal in the UV region is an experimental evidence for the presence of antiferromagnetic Fe-porphyrin correlations. The functional properties of hemoglobin such as the binding of O_2_, the Bohr effect and the cooperativity are explained based on the magnetic correlations. This analysis suggests that magnetism could be involved in the functioning of hemoglobin.

## Introduction

Pauling and Coryell showed that the magnetic susceptibility of deoxy-HbA exhibits a Curie-type (1/*T*) temperature dependence, while for oxy-HbA it is weakly negative implying that the total spin *S* = 0 for the molecule^1,2^. Mössbauer experiments^3^ are consistent with the view that Fe is in an *S* = 2 state in deoxy-HbA, while its magnetic moment is found to be $\lesssim 1\,{{\mu }}_{{\rm{B}}}$ in the oxy case. The magnetic circular dichroism (MCD) measurements^4^ find an anomalous line shape for the temperature-dependent MCD spectra in the UV region. The HbA molecule exhibits remarkable functional properties such as the cooperativity^5–7^ and the Bohr effect^8–11^, which enhance its oxygen carrying capacity. When one of the four Fe ions in HbA combines an oxygen molecule, the other three Fe ions attract oxygens cooperatively. The Bohr effect denotes the characteristic of HbA through which the oxygen affinity depends on the pH of the medium. Despite many years of study, there still remains open questions on the nature of the spin and charge distributions and the role of magnetism in the functioning of HbA and other heme-proteins^7,12,13^.

We study the electronic state of one heme group by using an effective multi-orbital Anderson impurity model^14,15^, which is described in the Methods section. The parameters of this model are obtained by the density functional theory^16^ (DFT) calculations, where we use the molecular coordinates determined by the X-ray measurements^17,18^. This way the stereochemical effects are included. In the DFT calculation of the Anderson model parameters, we use the Gaussian program^19^ with the BP86 energy functional (BP86)^20,21^ and the 6–31 G basis set. We then study this model with the quantum Monte Carlo (QMC) calculations by using the Hirsch-Fye algorithm^22^ while keeping all of the host states obtained by the DFT. However, the transverse component of the Hund’s coupling is not taken into account during the simulations. For the Coulomb interaction parameters we use *U* = 4 eV and *J* = 0.9 eV. This DFT + QMC approach is described in more detail in the Methods section and the Supplementary Information.

We note that the electronic state of the heme proteins and related molecules were previously studied by using the DFT^23^, multiconfigurational methods^24^, DFT + *U*^25,26^, and by combining the DFT with the dynamical mean field theory (DMFT)^27–30^. The DFT + DMFT method is similar to the DFT + QMC approach used here. However, there are differences. In the DFT + DMFT calculations the molecular coordinates are obtained computationally, while in the DFT + QMC approach we use those obtained by the X-ray measurements. In particular, the DFT + DMFT calculations were used to study the charge and spin state of the Fe site. On the other hand, we study the magnetic-moment formation in the host along with the Fe site, and also the Fe-host magnetic correlations. In addition, we analyze the available experimental data on the magnetic correlations along with the DFT + QMC results, and investigate possible consequences for the biological functioning of HbA.

**Figure 1:**
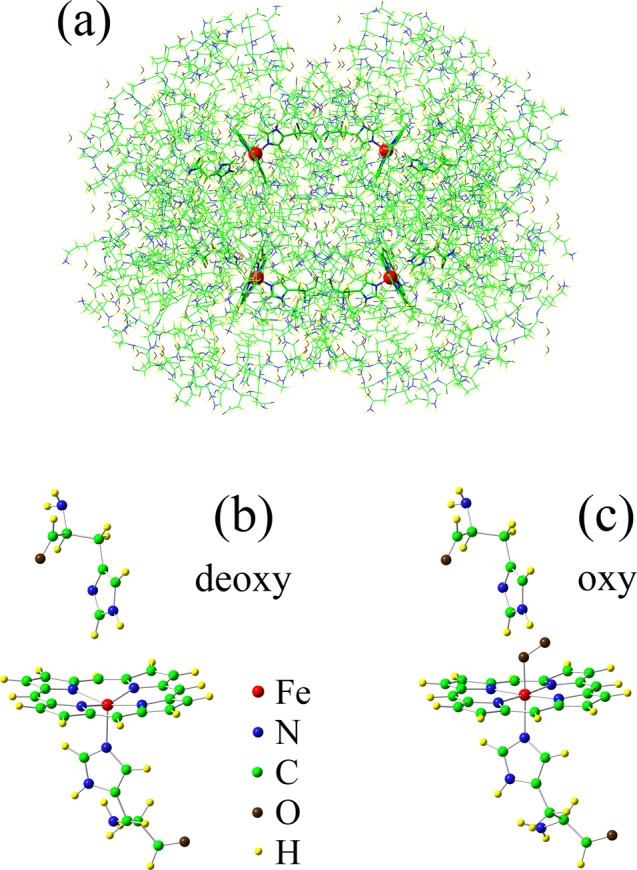
Molecular structure of HbA and the heme clusters. (**a**) Molecular structure of deoxy-HbA obtained by X-ray diffraction^18^ (Protein Data Bank, Keyword: 2DN2). Truncated (**b**) deoxy and (**c**) oxy-heme clusters which we have used in the DFT + QMC calculations. The way these clusters are obtained from the full HbA molecular structures is described in the Supplementary Information.

Figure 1(a) shows the molecular structure of deoxy-HbA^18^. We have performed our DFT + QMC calculations for the truncated clusters shown in Fig. 1(b,c).

**Figure 2:**
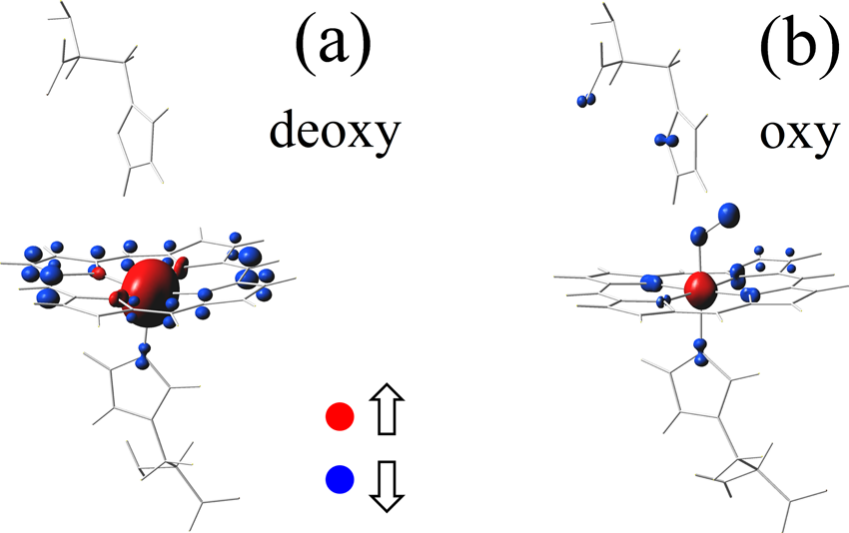
Magnetic-moment density. Illustration of the magnetic-moment density ***M***(**r**) for (**a**) the deoxy and (**b**) oxy-heme clusters at *T* = 150 K. Here, the red (blue) color indicates the atomic orbitals which have magnetic moments pointing up (down). The magnitude of ***M***(**r**) at an atomic site is proportional to the volume of the bubble at that site. The calculation of *M*(r) is described in the Supplementary Information.

Figure 2 shows DFT + QMC results for the magnetic-moment density ***M***(**r**) at temperature *T* = 150 K. In the deoxy case, we observe that the Fe site has a large up moment ≈4.6 *μ*_B_. The neighboring nitrogen sites have smaller moments, while at the carbon sites ***M***(**r**) points down. These down moments originate from the partially-occupied host states which consist of the C(2*p*_*z*_) orbitals. Hence, antiferromagnetic correlations exist between the large Fe magnetic moment and the host moments spread out in the porphyrin layer in deoxy-heme. In oxy-heme, the Fe moment is reduced, but remains finite. In addition, the neighboring O_2_ and N sites have magnetic moments which are antiferromagnetically coupled to that of Fe. In the oxy case, the antiferromagnetic screening cloud is more tightly localized around the Fe site. We note that the real-space structure of the magnetic correlations found by DFT + QMC are different than those found by GGA + *U*^26^. In the deoxy case, GGA + *U* does not obtain the antiferromagnetic Fe-porphyrin correlations. In the oxy case, GGA + *U* finds magnetic moment formation only at the Fe and the O_2_ sites. The DFT + QMC results show that, in the oxy case, magnetic moments also form in the porphyrin layer and in particular at the N sites neighboring Fe. Additional DFT + QMC data on the spin and charge distributions are presented in the Supplementary Information. The calculation of ***M***(**r**) is also described in the Supplementary Information.

**Figure 3:**
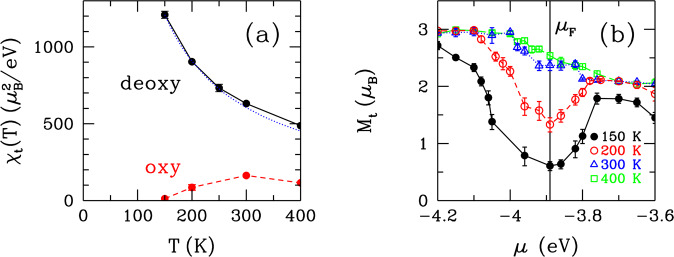
Spin susceptibility and the magnetic moment. (**a**) Temperature dependence of the total spin susceptibility *χ*_t_ for the deoxy and oxy-heme clusters. The blue dotted curve denotes the Curie (1/*T*) temperature dependence. (**b**) Total magnetic moment *M*_t_ versus the chemical potential *μ* near the Fermi level *μ*_F_ at various temperatures for the oxy-heme cluster. Here, the black vertical line denotes *μ*_F_ at *T* = 150 K.

Figure 3(a) shows the temperature dependence of the total spin susceptibility *χ*_t_. For the deoxy-heme cluster, *χ*_t_ follows a nearly-perfect Curie *T*-dependence. The total effective magnetic moment ***M***_**t**_ is ≈4.1 *μ*_B_ at *T* = 150 K, which is reduced from that of Fe due to the Fe-porphyrin antiferromagnetic correlations. In the oxy case, there are two temperature regimes separated by a crossover temperature T^*^ ≈ 300 K: In the high-*T* regime, *T* > T^*^, *χ*_t_ has a Curie-type *T* dependence with an effective total moment ≈2.1 *μ*_B_. In the low-*T* regime, *T* < T^*^, *χ*_t_ decreases with decreasing *T*. When the QMC finite-Δ*τ* effects are taken into account, this decrease becomes more rapid as shown in the Supplementary Fig. S4. For *T* < 300 K, the reduction of *χ*_t_ with respect to that of deoxy-heme is mainly due to the collapse of the Fe magnetic moment because of the loss of the ferromagnetic correlations among the Fe(3*d*_*v*_) orbitals. For *T* < 300 K, *χ*_t_ is reduced due to the suppression of magnetism at the Fermi level. This is seen in Fig. 3(b), which shows that the total moment ***M***_**t**_ gets suppressed within ≈0.15 eV of the Fermi level as *T* decreases. In Supplementary Fig. S1(b), the total spin susceptibility *χ*_t_ is plotted as a function of *μ*, where the suppression of *χ*_t_ at the Fermi level is seen. In order to study the susceptibility as a function of the real frequency, it would be necessary to carry out a maximum-entropy analytic continuation, which requires QMC data with very good statistics. Nevertheless, by simply plotting *χ*_t_ or ***M***_**t**_ as a function of *μ* at the Fermi level, it is possible to obtain a characteristic energy scale for the suppression of magnetism. We attribute this suppression to the transfer of electrons from mainly the O_2_ to the Fe(3*d*_*v*_) orbitals, in particular, to the so-called *t*_2*g*_ orbitals, *v* = *xy*, *xz* and *yz*. These are discussed further in the Supplementary Information.

Experimentally, the spin susceptibility of oxy-HbA vanishes at room temperature^1,31^, while we find that *χ*_t_ ≈ 150 ${\mu }_{{\rm{B}}}^{2}/$eV per heme. It is possible that because of the various approximations, such as the neglect of the transverse component of the Hund’s coupling or the temperature dependence of the molecular coordinates and the use of the reduced heme-clusters, the DFT + QMC approach is underestimating the value of T^*^. If this is indeed the case, then it will have consequences for the binding mechanism of O_2_ to Fe in heme and the Bohr effect: We note that, in general, charge transfer to the Fe(3*d*) orbitals would be energetically costly because of the large Coulomb repulsion over there. However, as seen in the Supplementary Information, in heme this is overcome by making use of the the upper-Hubbard level of the Fe(3*d*_*xy*_) orbital. This is where the Fe(3*d*_*xy*_) orbital becomes doubly occupied, and it is located very close to the Fermi level. This turns out to be a critical feature of the electronic structure of oxy-heme, because when the chemical potential is away from this region, the magnetism is not suppressed. We think that, in order to minimize its energy, the system is developing magnetic moments and magnetic correlations by redistributing electrons. It is a real possibility that this gain in energy is responsible for the binding of O_2_ to heme. These suggest that the binding of O_2_ to Fe in heme has a magnetic origin. We note that the stereochemical effects^6^ are clearly important in O_2_ binding. We are proposing that the magnetism, which is controlled by the stereochemical effects, has the key role in the perfectly reversible binding of O_2_ to Fe in heme.

In the Bohr effect, the oxygen affinity of HbA is controlled by the hydrogen ion concentration in the red blood cells. We have seen above that the magnetism is suppressed within a narrow energy window at the Fermi level. Hence, the magnetic properties depend sensitively on the electron filling. Any changes which effectively moves the chemical potential away from this narrow region will affect the binding of O_2_. This could be how the pH influences the oxygen affinity. However, we note that a more realistic modelling of the Bohr effect would also require taking into account how pH affects the complex interactions between the medium and the HbA molecule^7^. Here, we consider only the initial and the final states of the O_2_ binding reaction. To the extent that the spin correlations dominate the O_2_ binding, we expect this discussion to be relevant for the actual process.

We note that the above suggested mechanism for the Fe-oxygen bonding may not be limited to heme, but may also be relevant for bonding in other compounds containing transition metals, for example the transition-metal oxides. This type of bonding is more complicated than the usual covalent or ionic bondings because it involves the upper-Hubbard level of the 3*d* orbitals and the magnetic correlations.

An interesting feature which emerges from these calculations is the antiferromagnetic coupling between Fe and the porphyrin layer. Experimental evidence for this is provided by the MCD data on deoxy-HbA in the UV region. The MCD intensity Δ*ε*(*E*) is the difference between the left-circularly polarized (LCP) and the right-circularly polarized (RCP) light absorption within an applied magnetic field parallel to the direction of light propagation. The MCD spectrum of deoxy-HbA has a peak in the UV region at ≈3 eV which has an anomalous line shape and a 1/*T* temperature-dependence^4^. The optical absorption *ε*(*E*) has a *T*-independent peak at the same energy. It is known that the optical absorption at ≈3 eV is due to transitions from the bonding *π* to the antibonding π^*^ states which consist of the C(2*p*_*z*_) orbitals of the porphyrin layer^4^. The composition of the *π* and π^*^ states are illustrated in the Supplementary Fig. S6. The MCD spectrum of deoxy-HbA in UV region is anomalous in the following sense: In the usual case, the *T*-dependent piece of the MCD spectrum first has a dip and then a peak as the frequency increases, whereas in deoxy-HbA the MCD spectrum first has a peak and then a shallow dip. Here, we show that the anomalous MCD signal is caused by the orbital-selective optical transitions from the occupied *π* orbitals to two partially occupied π^*^ orbitals, which we label as ${\pi }_{1}^{\ast }$ and ${\pi }_{2}^{\ast }$. According to the DFT + QMC calculations, the ${\pi }_{1}^{\ast }$ state is nearly half-filled while the ${\pi }_{2}^{\ast }$ is nearly empty, which is discussed further in the Supplementary Information. We note that this mechanism for the anomalous MCD spectrum of deoxy-HbA was originally proposed by Sharanov *et al*.^32,33^. Here, we observe that the DFT + QMC data reproduces the experimental data in agreement with the previous predictions. This is experimental evidence for the Fe-host antiferromagnetic correlations found in the DFT + QMC calculations.

**Figure 4:**
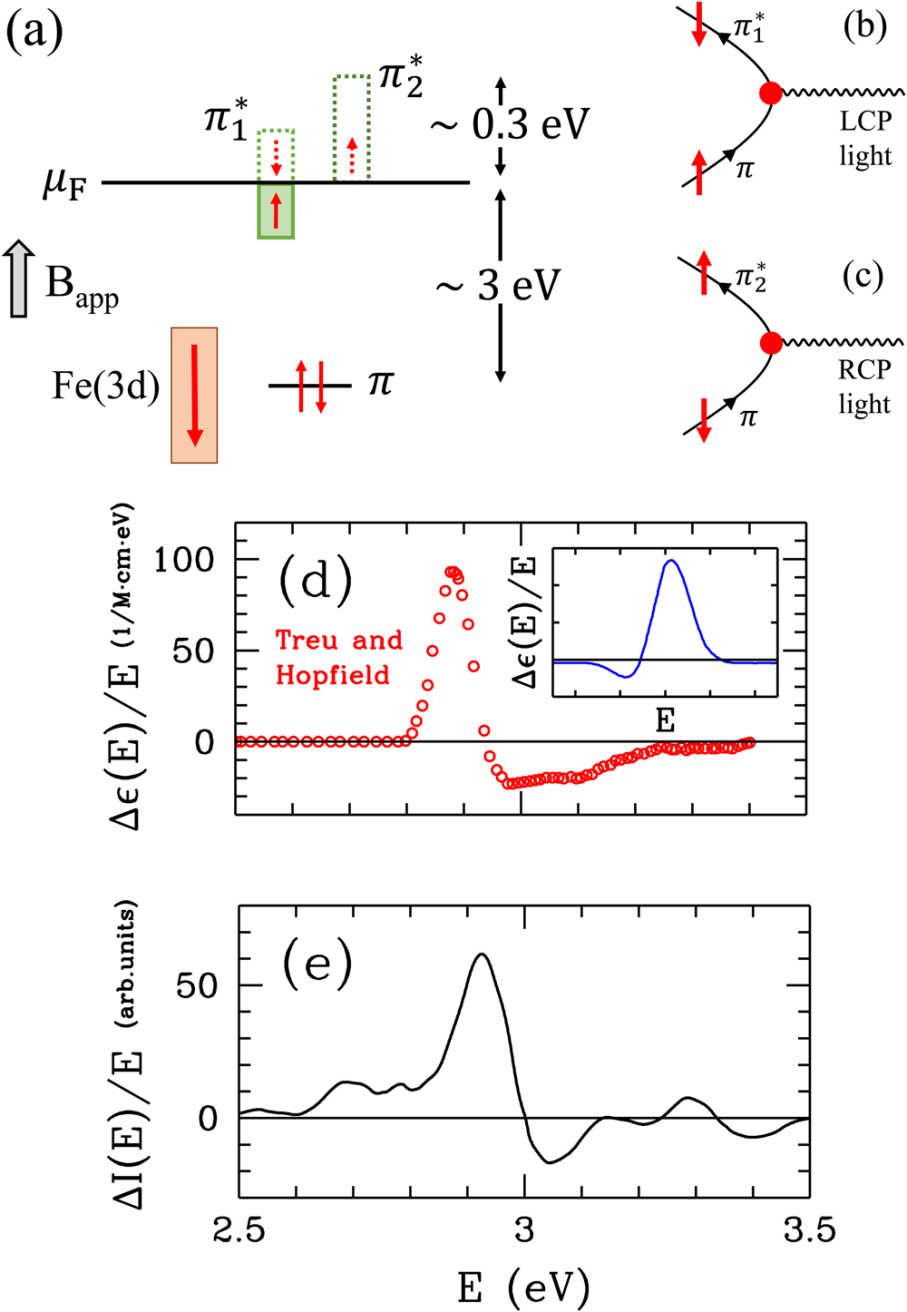
Anomalous MCD spectrum of deoxy-HbA in the UV region. (**a**) Illustration of the spin polarizations for the Fe(3*d*), and the bonding *π* and the antibonding ${\pi }_{1}^{\ast }$ and ${\pi }_{2}^{\ast }$ host states in an applied magnetic field **B**_app_ for deoxy-heme. For **B**_app_ pointing up, the total Fe(3*d*) spin gets polarized in the down direction, while the spin of the nearly half-filled ${\pi }_{1}^{\ast }$ state gets polarized parallel to the field. The ${\pi }_{2}^{\ast }$ state is nearly empty. From the DFT + QMC results we know that the *π* states are located about 3 eV below the Fermi level, and the widths of the ${\pi }_{1}^{\ast }$ and ${\pi }_{2}^{\ast }$ states are about 0.3 eV. (**b**) Feynman diagram representing the absorption of LCP light in the optical transition $\pi \to {\pi }_{1}^{\ast }$. Here, an up-spin electron in a *π* state makes a transition to the ${\pi }_{1}^{\ast }$ state becoming down-spin by absorbing LCP light (denoted by the wavy line). In (**c**), the $\pi \to {\pi }_{2}^{\ast }$ transition is shown for the absorption of RCP light, where a down-spin *π* electron makes a transition to the ${\pi }_{2}^{\ast }$ state becoming up-spin. Here, the red dot is the effective vertex for the optical transition. Even though there is spin-orbit interaction only at the Fe(3*d*) orbitals, the *π* states can gain an effective spin-orbit coupling through antiferromagnetic coupling and hybridization with the Fe(3*d*) orbitals, which are discussed in the Supplementary Information. (**d**) Anomalous MCD line shape in the UV region for deoxy-HbA from the experiments by Treu and Hopfield^4^. Inset: Line shape normally expected for the temperature-dependent MCD spectrum^34^. (**e**) Results from our calculation of the MCD spectrum for the deoxy-heme cluster.

As illustrated in Fig. 4(a), within an applied field **B**_app_ pointing up, the Fe spin will be polarized in the down direction. Meanwhile, the spin polarization of ${\pi }_{1}^{\ast }$ will be parallel to the field, because of the Fe-${\pi }_{1}^{\ast }$ antiferromagnetic correlations. Hence, during an LCP (RCP) optical transition from the *π* state, it will be energetically more favorable for the ${\pi }_{1}^{\ast }$ (${\pi }_{2}^{\ast }$) state to absorb a down-spin (up-spin) electron. These orbital-selective optical transitions are illustrated in Fig. 4(b,c). We have calculated the MCD spectrum due to these transitions as discussed in the Supplementary Information. Figure 4(d,e) present the experimental and the calculated MCD spectra, respectively. The inset of Fig. 4(d) illustrates the line shape normally expected for the *T*-dependent MCD spectrum^34^. The anomalous MCD line shape of deoxy-HbA seen in Fig. 4(d) had been attributed to a negative spin-orbit coupling^4^. We show that it instead originates from the antiferromagnetic coupling between the Fe(3*d*) and π^*^ states, as was previously suggested by Sharonov *et al*.^32,33^. Because of this, ${\pi }_{1}^{\ast }$ and ${\pi }_{2}^{\ast }$ porphyrin states act as if they have negative *g*-factors. The agreement with the experimental data can be improved further by incorporating into the analysis the optical absorption data as shown in the Supplementary Information.

Next, we compare the DFT + QMC results with the experimental magnetic susceptibility measurements. Assuming independent heme groups, the magnetic susceptibility measurements yield an effective magnetic moment per heme ${M}_{{\rm{heme}}}^{{\rm{expt}}}=5.46\,{\mu }_{{\rm{B}}}$ in deoxy-HbA^1^. Since this is larger than the spin *S* = 2 value of 4.9 *μ*_B_, Pauling and Coryell already pointed out the possibility of inter-heme ferromagnetic correlations^1^. These imply that the average inter-heme magnetic correlation $\langle {M}_{{\rm{heme}},1}^{z}{M}_{{\rm{heme}},2}^{z}\rangle \approx 1.9\,{\mu }_{{\rm{B}}}^{2}$ for deoxy-HbA. On the other hand, the DFT + QMC calculations find *M*_Fe_ ≈ 4.6 *μ*_B_ and ${M}_{{\rm{heme}}}\approx 4.1\,{\mu }_{{\rm{B}}}$, which is reduced from *M*_Fe_ because of the antiferromagnetic Fe-host coupling. This yields an average inter-heme magnetic correlation $\langle {M}_{{\rm{heme}},1}^{z}{M}_{{\rm{heme}},2}^{z}\rangle \approx 4.3\,{\mu }_{{\rm{B}}}^{2}$ for deoxy-HbA. Hence, the average inter-heme magnetic correlations are found to be stronger when the antiferromagnetic Fe-host coupling is taken into account. From comparison with the experimental data, we thus find evidence for the existence of significant inter-heme ferromagnetic correlations.

The cooperativity of HbA enhances its O_2_ carrying capacity. It arises from the property that the binding rate of O_2_ to HbA depends on how many of the four heme groups in HbA are already oxygenated. For example, the binding of the first O_2_ to HbA occurs at a rate much slower than that of the last (fourth) O_2_ to bind. Clearly, any discussion of cooperativity requires a multi-heme model. However, we will briefly comment on the implications of the DFT + QMC results. In particular, we suggest two possible scenarios on how the presence of inter-heme ferromagnetic correlations could lead to the cooperativity. In the first scenario, the first O_2_ to bind HbA needs to overcome the inter-heme ferromagnetic interactions, because it will break three of the inter-heme ferromagnetic bonds. Upon the binding of the first O_2_ to a heme group in HbA, the magnetic moment of that heme group vanishes. The following O_2_’s will bind more easily because now there are fewer inter-heme ferromagnetic bonds to break. A second alternative scenario is based on the spin non-conservancy in the binding of O_2_ to heme. We note that while O_2_ is in a triplet state (*S* = 1) and deoxy-heme is considered to be *S* = 2, the resultant oxy-heme is in an *S* = 0 state, hence the spin is not conserved in the binding of O_2_ to a heme group. This non-conservancy of the total spin may be limiting the reaction rate for O_2_ binding^23^. However, if there indeed exist sufficiently strong inter-heme ferromagnetic correlations, then spin transfer may be possible from one heme group to another within HbA. Hence, when the O_2_’s bind cooperatively, the total spin can be conserved by inter-heme spin transfer. This could eliminate the limit on the O_2_ binding rate arising from the spin non-conservancy. According to this scenario, we expect the cooperative (simultaneous) binding of four O_2_’s to HbA to occur faster than the binding of the first O_2_ to HbA. Clearly, these scenarios are only speculative ideas at the moment. It would be necessary to study multi-heme models in order to test them.

We have shown that magnetic moment formation and magnetic correlations are key electronic properties of deoxy and oxy-HbA. It is remarkable that these properties, which arise from the strongly-correlated electrons, could play a role in the functioning of hemoglobin. We note that there are a large number of metalloproteins, metalloenzymes and other bioinorganic molecules containing transition-metal centers^35^. Hence, we suggest that the magnetic effects could have a general role in the functioning of bioinorganic molecules with a distinct place in the emerging field of quantum biology.

## Methods

### Effective Anderson impurity model for heme

We use an effective multi-orbital Anderson impurity model^14,15^, where the five Fe(3*d*) orbitals are taken as the impurity states and the remaining orbitals are treated as the host states, to describe the electronic properties of the deoxy and oxy-heme clusters. The multi-orbital Anderson Hamiltonian with the intra and inter-orbital Coulomb interactions is given by

1 $$\begin{array}{ccc}H & = & \sum _{m,\sigma }\,({\varepsilon }_{m}-\mu ){c}_{m\sigma }^{\dagger }{c}_{m\sigma }+\sum _{\nu ,\sigma }({\varepsilon }_{d\nu }-\mu ){d}_{\nu \sigma }^{\dagger }{d}_{\nu \sigma }+\sum _{m,\nu ,\sigma }\,({V}_{m\nu }{c}_{m\sigma }^{\dagger }{d}_{\nu \sigma }+{V}_{m\nu }^{\ast }{d}_{\nu \sigma }^{\dagger }{c}_{m\sigma })\\  &  & +\,\sum _{\nu }\,U{n}_{\nu \uparrow }{n}_{\nu \downarrow }+\sum _{\nu  > \nu {\rm{{\prime} }},\sigma }\,({U}^{{\rm{{\prime} }}}{n}_{\nu \sigma }{n}_{\nu {\rm{{\prime} }}-\sigma }+({U}^{{\rm{{\prime} }}}-J){n}_{\nu \sigma }{n}_{\nu {\rm{{\prime} }}\sigma }),\end{array}$$

where ${c}_{m\sigma }^{\dagger }$ (*c*_*mσ*_) operator creates (annihilates) an electron in the *m*’th host state with spin *σ*, and ${d}_{\nu \sigma }^{\dagger }$ (*d*_*vσ*_) is the creation (annihilation) operator for a localized electron with spin *σ* at the Fe(3*d*_*v*_) orbital. The electron occupation operator for the Fe(3*d*_*v*_) orbitals is ${n}_{\nu \sigma }={d}_{\nu \sigma }^{\dagger }{d}_{\nu \sigma }$. The energies of the host and the Fe(3*d*_*v*_) states are *ε*_*m*_ and *ε*_*dv*_, respectively. The hybridization matrix element between the *m*’th host state and the Fe(3*d*_*v*_) orbital is *V*_*mv*_. The intra-orbital Coulomb repulsion is *U*, while *U*′ and *U*″ = *U*′ − *J* are the Coulomb interactions between two 3*d* electrons in different orbitals with antiparallel and parallel spins, respectively. Here, *J* is the ferromagnetic Hund’s coupling constant. In the case of a free atom, the relation *U*′ = *U* − 2*J* holds, which we also use here. The chemical potential *μ* is introduced because the QMC calculations are performed at finite temperatures in the grand canonical ensemble by using the Hirsch-Fye algorithm^22^. At each value of the temperature, we adjust *μ* so that the cluster has the correct number of electrons, which is discussed in Supplementary Fig. S3. We obtain the values of *ε*_*m*_, *ε*_*dv*_ and *V*_*mv*_ by the density-functional theory (DFT)^16^. The DFT calculations are carried out by using the Gaussian program^19^ with the BP86 energy functional^20,21^ and the 6–31 G basis set with 483 basis functions for the deoxy-heme cluster and 501 basis functions for the oxy-heme cluster. Further information on this procedure is given in the Supplementary Information. For the interaction parameters we use *U* = 4 eV and *J* = 0.9 eV. A similar approach, which also uses a maximally-localized single-particle basis, was introduced by Ref. ^36^ for mapping the electronic state of molecular nanomagnets onto strongly correlated models.

We have performed the DFT + QMC calculations for clusters obtained by truncating the full deoxy and oxy-heme molecular structures from the Protein Data Bank as described in the Supplementary Information. For understanding the functioning of HbA, the role of the stereochemical effects have been investigated. In particular, it has been emphasized that Fe moves by about 0.4 Å towards the porphyrin ring upon O_2_ binding^17^. Since we are using coordinates determined by the X-ray measurements, these stereochemical effects are already included in our model.

In Eq. (1), we include the longitudinal component of the Hund’s interaction, however the transverse component, which consists of the spin-flip and the pair-hopping terms, is not included, because it cannot be treated with the Hirsch-Fye algorithm. In addition, we neglect the temperature dependence of the molecular coordinates. In spite of these approximations, the DFT + QMC technique applied to this effective impurity model offers a realistic treatment for the electronic state of HbA. We note that these DFT + QMC calculations represent the only computational approach which yields agreement with the magnetic susceptibility, Mössbauer, and the MCD data on HbA at the same time.

## Supplementary information

Magnetic mechanism for the biological functioning of hemoglobin Supplementary Information.

## Data Availability

The data that support the findings of this study are available from the corresponding author upon reasonable request.
