# Supplementary material for: Magnetic mechanism for the biological functioning of hemoglobin
Source: Sci Rep. 2020 May 22;10:8569. doi: 10.1038/s41598-020-64364-y (PMC7244595; doi:10.1038/s41598-020-64364-y)
Supplement: Supplementary file 1 — Magnetic mechanism for the biological functioning of hemoglobin Supplementary Information. [file 41598_2020_64364_MOESM1_ESM.pdf]

Magnetic mechanism for the biological functioning of hemoglobin
Supplementary Information

Selma Mayda[1,2], Zafer Kandemir[1], Nejat Bulut[1,*], and Sadamichi Maekawa[3,4]

[1]Department of Physics, Izmir Institute of Technology, Urla 35430, Turkey
[2]Department of Materials Science and Engineering, Izmir Institute of Technology, Urla 35430, Turkey
[3]RIKEN Center for Emergent Matter Science, Wako 351-0198, Japan
[4]Kavli Institute for Theoretical Sciences, University of Chinese Academy of Sciences, Beijing 100049, China
[*]nejatbulut@iyte.edu.tr

Calculation of the Anderson model parameters with DFT for heme clusters

We obtain the one-electron parameters ε_m, $\varepsilon_{d\nu}$ and $V_{m\nu}$ of the effective Anderson model by making use of the DFT method. These calculations are performed for a reduced cluster with molecular coordinates obtained from the Protein Data Bank. In this calculation, instead of the atomic orbitals, we use the natural atomic orbitals (NAO's)[1], which form an orthonormal and maximally-localized single-particle basis. First, we express the Fock matrix in the NAO basis. We take the Fe$(3d_\nu)$ NAO's as the impurity orbitals and their energy levels as $\varepsilon_{d\nu}$'s in the Anderson Hamiltonian. Diagonalizing the remaining part of the Fock matrix, we obtain the host eigenstates $|u_m\rangle$ and their energy levels ε_m and the hybridization matrix elements $V_{m\nu}$. This procedure is explained in more detail in Ref.[2–4]. The DFT calculations are carried out by using the Gaussian program[5] with the BP86 energy functional[6,7] and the 6-31G basis set with 483 basis functions for the deoxy-heme cluster and 501 basis functions for the oxy-heme cluster. We use ε_m, $\varepsilon_{d\nu}$ and $V_{m\nu}$ determined this way as input parameters for the QMC simulations. Our procedure for obtaining the Anderson model parameters differs from that in Ref.[8], because we are using the Gaussian program with the NAO basis set. However, it is similar to that presented by Ref.[9], which also uses a maximally-localized single-particle basis. During the QMC simulations, we use 476 host states for the deoxy cluster and 496 host states for the oxy cluster in addition to the 5 Fe(3d) impurity orbitals. Hence, all of the host states obtained by DFT are taken into account during the QMC simulations. For the Coulomb interaction parameters we use $U = 4$ eV and $J = 0.9$ eV. The dependence of the DFT+QMC results on the value of J will be discussed below in Supplementary Fig. S5.

Truncated heme clusters and the finite-size effects

The deoxy-HbA molecule seen in Fig. 1(a) contains four inequivalent heme groups, $\alpha_1 - \alpha_2 - \beta_1 - \beta_2$. The nearest-neighbor Fe-Fe distance varies between 34 Å and 39.5 Å. Since this molecule consists of about 9700 atoms, we performed our calculations for a reduced heme cluster obtained from the α_1 group, which is shown in Fig. 1(b). This cluster contains the porphyrin ring with Fe at the center, located below porphyrin is the distal histidine with the imidazole part, and also located at the top is the proximal histidine. It is thought that the proximal histidine is necessary for the stability of O_2 binding[10]. In obtaining this truncated cluster, we have replaced the methyl, vinyl and propionate side groups of porphyrin with hydrogen atoms. We have determined the coordinates of these substituted hydrogens by DFT optimization. The deoxy-heme cluster obtained this way consists of 75 atoms and 334 electrons ($C_{32}H_{30}FeN_{10}O_2$). In order to obtain the oxy-heme cluster, we have again started with the molecular structure of oxy-HbA determined by X-ray measurements from the Protein Data Bank (Keyword: 2DN1). From the α_1 heme group we have obtained the cluster seen in Fig. 1(c) with 77 atoms and 350 electrons ($C_{32}H_{30}FeN_{10}O_4$).

In obtaining the energy spectrum of the cluster with DFT, it is necessary to take into account the finite size effects arising from the boundary of the cluster. Even though the HbA molecule contains about 9700 atoms, we retain only 75 atoms for the deoxy-heme cluster shown in Fig. 1(b). In this case we find that a host state which is localized on the oxygen and carbon sites at the boundary of the cluster (at the lower edge of the cluster shown in Fig. 1(b)) has an energy close to the Fermi level. However, when we use larger clusters containing 87 or 96 atoms so that the distal histidine part contains more sites and the oxygen site is not close to the boundary, we find that this host state arising from the boundary moves away from the Fermi level to higher energies. Hence, for the 75 site cluster we have by hand removed the boundary host state in order to control the finite size effects.

Additional DFT+QMC data on the spin susceptibility

In Supplementary Fig. S1(a), we compare the total spin susceptibility of the cluster χ_t with the Fe$(3d)$ susceptibility χ_{Fe}. For the deoxy case, we see that χ_t is reduced with respect to χ_{Fe} due to the Fe-porphyrin antiferromagnetic coupling. Here, we also observe that χ_{Fe} obeys a perfect Curie T-dependence. In the oxy case, there is little difference between χ_t and χ_{Fe}. Both

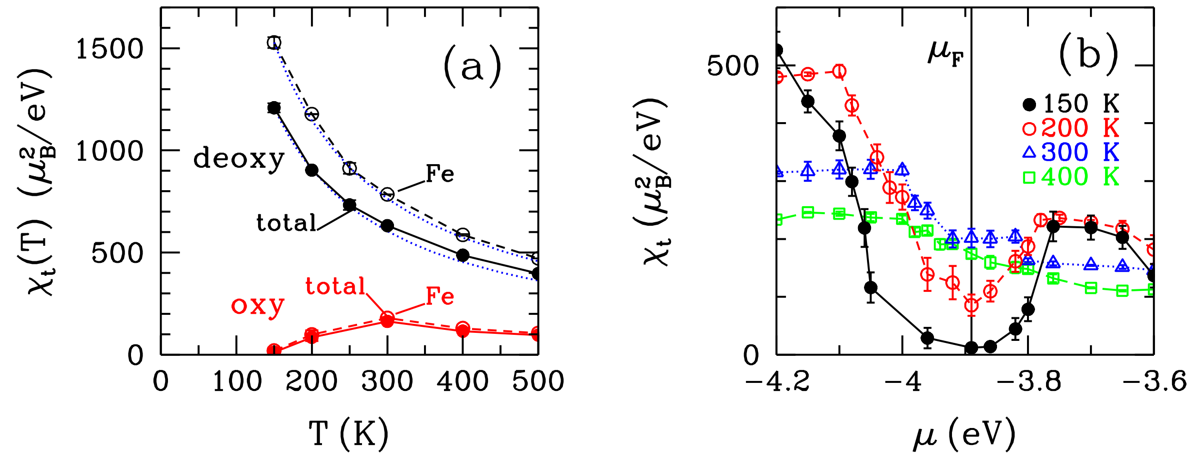

Supplementary Fig. S1 Additional DFT+QMC results on the spin susceptibility

(a) Total spin susceptibility χ_t of the deoxy and oxy-heme clusters denoted by the filled circles plotted as a function of the temperature. The results on the Fe spin susceptibility χ_{Fe} denoted by the open circles are also shown. Here, the blue dotted lines denote the $1/T$ temperature dependence. (b) χ_t versus the chemical potential μ near the Fermi level μ_F at various temperatures. Here, the black vertical line denotes μ_F at $T = 150$ K.

increase as $1/T$ for T down to 300 K. As T decreases below 300 K, χ_{Fe} and χ_t decrease.

In Supplementary Fig. S1(b), χ_t of the oxy-heme cluster is shown as a function of μ for various T. Here, we observe the suppression of χ_t within ≈ 0.15 eV of the Fermi level at low T.

Additional DFT+QMC data on the O_2 to Fe charge transfer and the magnetic gap in oxy-heme

In Supplementary Fig. S2(a), we see that the total electron number in the Fe($3d$) orbitals $\langle n_{3d} \rangle$ develops a peak at the Fermi level $\mu_F \approx -3.9$ eV at low T. Supplementary Fig. S2(b) shows $\langle n_\nu \rangle$ versus μ for the five Fe($3d_\nu$) orbitals. We observe that, if μ is kept fixed at -3.9 eV as T is lowered from 400 K to 200 K, then $\langle n_\nu \rangle$ for the t_{2g} orbitals ($\nu = xy$, xz, and yz) gets enhanced. In this case, the $3d_{xy}$ orbital has the largest increase. Meanwhile, for the e_g orbitals ($\nu = 3z^2 - r^2$ and $x^2 - y^2$), $\langle n_\nu \rangle$ gets suppressed. These show that the $3d$ orbitals are strongly coupled to each other as well as to their environment. We also observe that the upper Hubbard level of the $3d_{xy}$ orbital shifts down in energy with decreasing T. We are able to capture these effects because we treat Fe in heme as a Hund's impurity in the special electronic environment of oxy-heme by applying DFT+QMC. We think that the system is minimizing its energy by redistributing the electrons and developing magnetic correlations. These lead us to the notion that the binding of O_2 to Fe in heme is related to magnetism and the $3d$ Coulomb interactions.

In Supplementary Fig. S2(c), we see that, as the peak in $\langle n_{3d} \rangle$ develops, the total $3d$ magnetic moment M_{3d} gets suppressed at the Fermi level. At low T, M_{3d} approaches 1.65 μ_B, which is close to the spin-1/2 value.

The electrons transferred to the Fe($3d$) orbitals come mostly from a host state which we label as the $m = 171$'th host state. This state consists of the atomic orbitals from O_2 and the proximal histidine. Its wave function is illustrated in Supplementary Fig. S2(d). The electron occupation $\langle n_m \rangle$ of this state gets suppressed near the Fermi level at low temperatures, while its magnetic moment M_m gets enhanced reaching 0.6 μ_B at 150 K as shown in Supplementary Figs. S2(e) and (f), respectively.

Shift of the Fermi level with the temperature in oxy-heme

In Supplementary Fig. S3, we show results on the total electron number $\langle n_t \rangle$ versus μ for the oxy-heme cluster, which has 77 atoms and 350 electrons. We see that at 300 K the Fermi level $\mu_F \approx -3.8$ eV, while at $T = 150$ K we have $\mu_F \approx -3.9$ eV.

Finally, we note that, for $T = 150$ K, $\langle n_t \rangle$ does not change between $\mu_F \approx -3.9$ eV and -3.8 eV. However, the magnetism changes significantly between these two points.

QMC finite-$\Delta\tau$ effects in the oxy-heme case

The Matsubara-time step $\Delta\tau$ is introduced in the QMC method by dividing the inverse temperature $\beta = 1/T$ into L Matsubara-time slices, $\beta = L\Delta\tau$. The QMC results become exact in the limit $\Delta\tau \to 0$. All of the DFT+QMC results shown in the above figures were taken by using $\Delta\tau$ values between 0.125 and 0.2 eV^{-1}. In Supplementary Figures S4(a)-(d) we show the effects of using finite $\Delta\tau$ on $\langle n_t \rangle$, $\langle n_{3d} \rangle$, χ_t and M_t, respectively.

The charge-neutral oxy-heme cluster has 350 electrons. Supplementary Fig. S4(a) shows that $\langle n_t \rangle$ approaches 350.0 in the limit $\Delta\tau \to 0$ at $T = 150$ K. This result was obtained by using $\mu = -3.89$ eV. Hence, in the limit $\Delta\tau \to 0$ and at $T = 150$ K we have $\mu_F \approx -3.9$ eV. For 200 K, $\mu = -3.89$ eV was also used, while at $T = 300$ K we used $\mu = -3.8$ eV. These show that μ_F shifts from ≈ -3.8 eV at 300 K to ≈ -3.9 eV at 150 K.

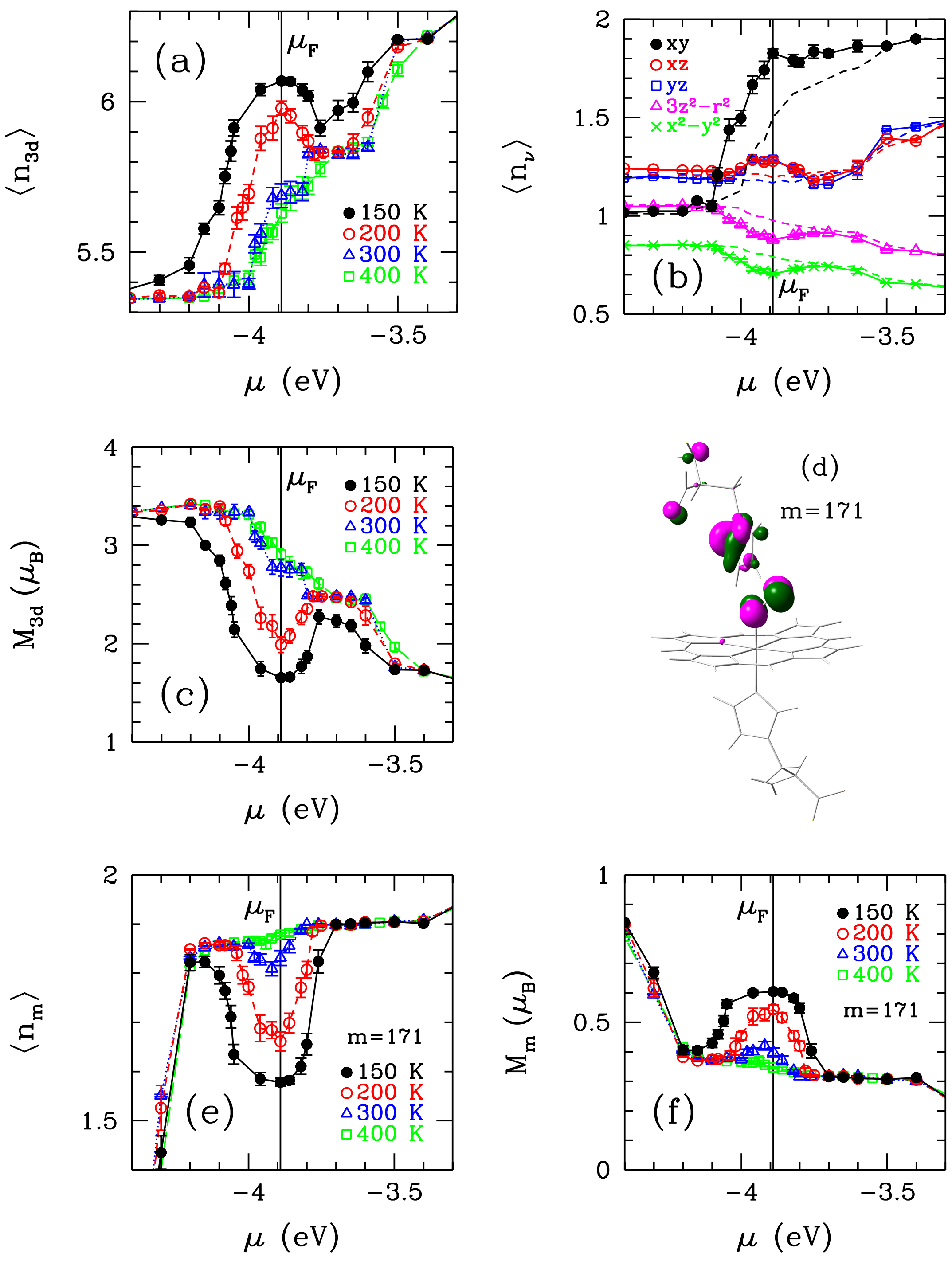

Supplementary Fig. S2 Additional DFT+QMC data on the charge transfer from O_2 to Fe in oxy-heme

(a) Total number of the electrons in the Fe(3d) orbitals $\langle n_{3d} \rangle$ plotted as a function of the chemical potential μ at various temperatures. (b) Electron occupation number of the Fe(3d_ν) orbitals $\langle n_\nu \rangle$ versus μ. Here, the data points connected by the solid lines denote $\langle n_\nu \rangle$ obtained at 200 K, while the dashed curves denote results obtained at 400 K. (c) Total magnetic moment of the Fe(3d) orbitals M_{3d} versus μ. (d) Illustration of the wave function of the $m = 171$'th host state, which is real valued. Here, the magenta and green colors denote the positive and the negative regions. (e) Electron number of the $m = 171$'th host state $\langle n_m \rangle$ versus μ. (f) Magnetic moment of the $m = 171$'th host state M_m versus μ. In these figures, the black vertical line denotes the Fermi level μ_F at $T = 150$ K.

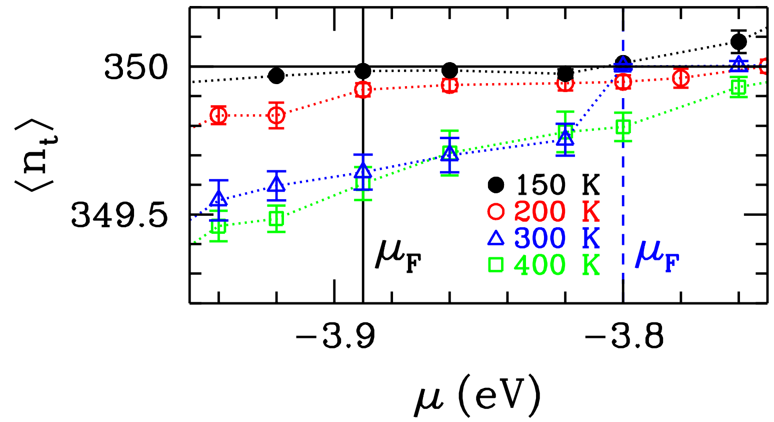

Supplementary Fig. S3 Shift of the Fermi level with the temperature for oxy-heme

Total electron number of the oxy-heme cluster $\langle n_t \rangle$ versus the chemical potential μ at various temperatures. The charge-neutral oxy-heme cluster has 350 electrons. We see that, as the temperature is lowered, the Fermi level μ_F shifts from ≈ -3.8 eV at 300 K to ≈ -3.9 eV at 150 K.

In Supplementary Fig. S4(b), we see that, in the limit $\Delta\tau \to 0$, $\langle n_{3d} \rangle$ increases from 5.9 to approximately 6.1 as T decreases from 300 K to 150 K. Hence, Fe in oxy-heme is in a ferrous state.

Supplementary Fig. S4(c) shows that $\chi_t \to 150\,\mu_B^2$ as $\Delta\tau \to 0$ at 300 K, while for 200 K we see that χ_t extrapolates to a much reduced value. Hence, the magnetic gap is already developed at 200 K. When the $\Delta\tau \to 0$ corrections are taken into account, the drop in χ_t for $T < 300$K, which was seen in Fig. 3(a), becomes more rapid. Similarly, in Supplementary Fig. S4(d) we observe a rapid drop in M_t in the limit $\Delta\tau \to 0$ as T decreases. These figures show that the finite-$\Delta\tau$ effects do not change our conclusions.

Dependence of the magnetism on the Hund's coupling J

In the Supplementary Fig. S5, we plot the effective magnetic moment of the Fe($3d$) orbitals M_{3d} as a function of the Hund's coupling J for the deoxy and oxy clusters at $T = 700$ K. While for the deoxy case, M_{3d} exhibits a smooth J dependence, we observe a jump between 1.0 and 1.1 eV in the oxy case, which means that the high-spin state is becoming stable when J is increased from 1.0 to 1.1. eV. This J dependence resembles that found for the molecule $Fe(phen)_2(NCS)_2$ by the DFT+DMFT calculations[11], where the high-spin state is found to become stable as J increases from 0.85 to 0.9 eV. We think that the crossover values of J are different because the oxy-HbA we are studying and the molecule $Fe(phen)_2(NCS)_2$ have different structures and bond lengths, hence resulting in different hybridization parameters.

Calculation of the MCD spectrum in the UV region for deoxy-heme

Supplementary Fig. S6(a) illustrates the initial, intermediate and the final states in the $\pi \to \pi_1^*$ optical transition with LCP light absorption when the applied field is in the up direction. The left panel shows the initial state where the Fe($3d_\nu$) orbital, which is hybridizing with the bonding π state, has a down-spin electron, while the π state is doubly occupied. Because of the Fe($3d$)-π^* antiferromagnetic correlations, the antibonding π_1^* state has an up-spin electron. In the intermediate state (middle panel), we see that, upon LCP light absorption, the down-spin π electron moves to the π_1^* state, while the up-spin π electron spin-flips together with the Fe($3d$) down-spin electron through antiferromagnetic exchange. In this intermediate state, the up-spin Fe($3d$) electron is in an excited state because of the ferromagnetic Hund's coupling to the other Fe($3d$) spins. Hence, it spin-flips one more time by making use of the spin-orbit coupling at the Fe site, which leads to the final state (right panel). There is a similar process for the $\pi \to \pi_2^*$ transition with the RCP light absorption. These optical transitions are orbital selective because of the antiferromagnetic Fe-π^* correlations, which leads to the anomalous MCD line shape.

From comparisons with the optical absorption data, which will be shown below in Supplementary Fig. S8, we deduce that a bonding π state located at -5.6 eV, which we label as π_1, is dominant in the UV optical processes. In fact, we find that the leading contribution to UV MCD arises from the $\pi_1 \to \pi_1^*$ transition for the LCP light absorption, and $\pi_1 \to \pi_2^*$ for RCP absorption. Supplementary Figs. S6(b)-(d) show the wave functions of these π_1, π_1^*, and π_2^* states, which consist of the C($2p_z$) atomic orbitals of the porphyrin layer.

Even though the π states do not have significant spin-orbit coupling, they can gain an effective coupling because of antiferromagnetic correlations and hybridization with the Fe($3d$) orbitals. Supplementary Figs. S6(e)-(f) illustrate the lowest-order process where a π state can gain an effective spin-orbit coupling. We note that the largest hybridization matrix elements of the π_1^* and π_2^* states are with the $3d_{xz}$ and $3d_{yz}$ orbitals, respectively, and they are both about 0.3 eV.

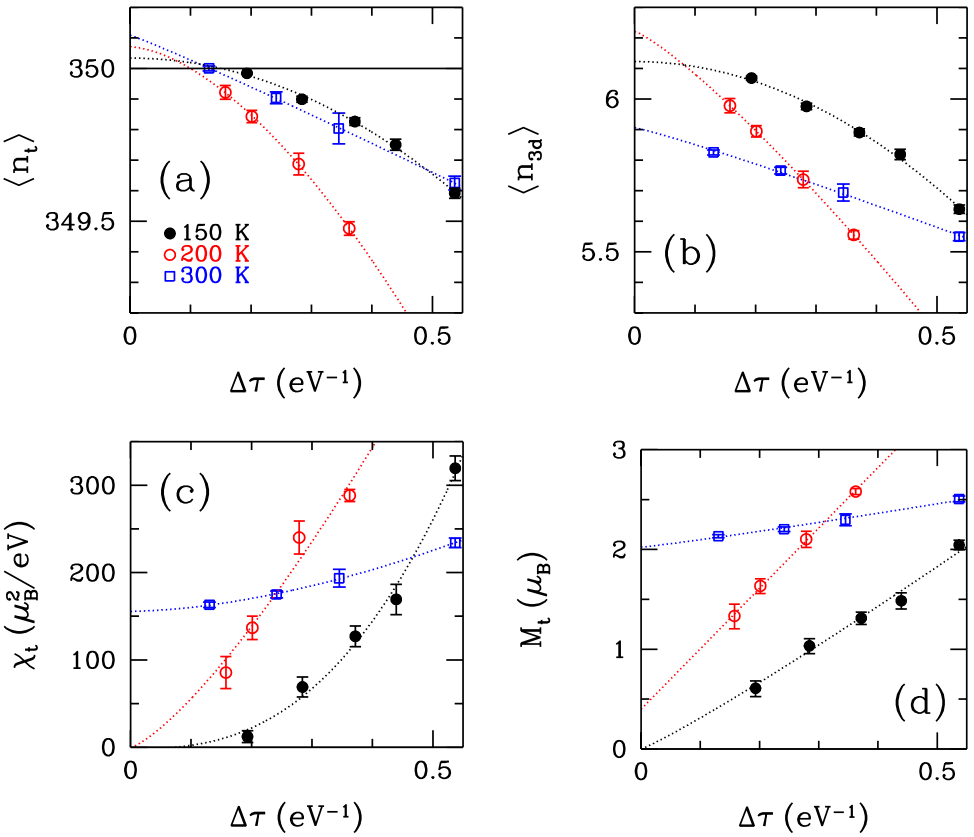

Supplementary Fig. S4 QMC finite-$\Delta\tau$ effects in the oxy-heme case

(a) Total number of the electrons in the cluster $\langle n_t \rangle$, (b) the total electron number in the Fe($3d$) orbitals $\langle n_{3d} \rangle$, (c) the total spin susceptibility χ_t, and (d) the total magnetic moment M_t plotted as a function of $\Delta\tau$ for the various values of T shown in (a). Here, $\Delta\tau$ is the Matsubara-time step used in the QMC simulations. The dotted curves are the least-squares fits to the QMC data.

We use the following simple expression for the T-dependent MCD spectrum in the UV region

$$\Delta I(E) = \sum_m \left(C_1 \, |\langle \pi_1^* | \mathbf{p} | m \rangle|^2 A_{\pi_1^*}(\varepsilon_m + E) - C_2 \, |\langle \pi_2^* | \mathbf{p} | m \rangle|^2 A_{\pi_2^*}(\varepsilon_m + E) \right) f(\varepsilon_m)(1 - f(\varepsilon_m + E)), \quad (1)$$

which is due to LCP $\pi \to \pi_1^*$ and RCP $\pi \to \pi_2^*$ optical transitions. Here, m sums over all of the bonding π host states, and C_1 and C_2 are coefficients to be determined. In addition, we use the matrix element $|\langle m' | \mathbf{p} | m \rangle|^2$, instead of $|\langle m' | p_- | m \rangle|^2$ or $|\langle m' | p_+ | m \rangle|^2$ where $p_\pm = p_x \pm i p_y$, since the experiments were performed using samples in solution.

In Supplementary Fig. S7(a)-(d), we show QMC data on the electronic properties of the π_1^* and π_2^* host states. Their spectral weights $A(\omega)$ are obtained in an approximate way by taking the derivative with respect to μ of their electron occupations $\langle n \rangle$ shown in Supplementary Fig. S7(a). The resulting $A_{\pi_1^*}(\omega)$ and $A_{\pi_2^*}(\omega)$ are shown in Supplementary Fig. S7(b). These spectral weights have peaks split by 0.2 eV corresponding to $2J_{\rm AF}$, where $J_{\rm AF} \sim 4|V_{\pi^*,\nu}|^2/U \sim 0.1\,eV$ is the maximum value of the Fe($3d_\nu$)-π^* antiferromagnetic exchange constant. Supplementary Fig. S7(c) shows $\langle (M^z)^2 \rangle$ versus μ for π_1^* and π_2^*. Supplementary Fig. S7(d) shows the function $\langle M_{3d}^z M_m^z \rangle$ between Fe($3d$) and the $m = \pi_1^*$ and π_2^* states. Here, we clearly see the Fe($3d$)-π^* antiferromagnetic correlations.

In Eq. (2) we take the ratio C_1/C_2 to be 4. This is the only fitting parameter we use in obtaining the spectrum shown in Fig. 4(e). Since π_1^* is already occupied by one up-spin electron and π_2^* is nearly empty, we expect the coefficient C_1 to be larger than C_2. Finally, since the polarizability of the total Fe($3d$) spin has $1/T$ temperature dependence, our result for the MCD intensity also exhibits this type T-dependence.

Comparison of the MCD and the optical absorption data in the UV region for deoxy heme

Supplementary Fig. S8(a) shows experimental data on the optical absorption of deoxy-heme[12]. The peak near 3 eV originates from $\pi \to \pi^*$ transitions. This peak has an asymmetrical line shape, where the intensity below 2.8 eV is much reduced compared

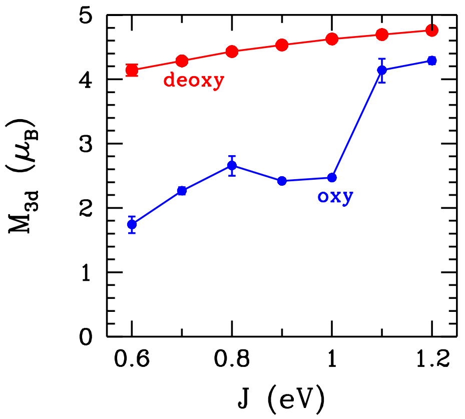

Supplementary Fig. S5 Dependence of M_{3d} on the Hund's coupling J

Total magnetic moment of the Fe($3d$) orbitals M_{3d} versus the Hund's coupling J for the deoxy and oxy-heme clusters at $T = 700$ K. We note that in the DFT+QMC calculations the molecular coordinates are determined according to the X-ray measurements taken at 300 K, and they are assumed to have no temperature dependence.

to the high energy tail.

A simple estimate of the optical absorption in the UV region is given by

$$I(E) = \sum_{m',m} |\langle m'|\mathbf{p}|m\rangle|^2 f(\varepsilon_m)(1 - f(\varepsilon_{m'}))\delta(E - (\varepsilon_{m'} - \varepsilon_m)), \tag{2}$$

where m and m' sum over the bonding π and antibonding π^* host states, respectively, and $\mathbf{p} = \sum_i (-e)\mathbf{r}_i$ is the operator for the electric-dipole moment of the cluster. Here, for ε_m we have used the DFT results, since the Coulomb interactions taken into account by QMC do not produce a significant shift in the doubly-occupied π states. The results for $I(E)/E$ are shown as the black bars for each transition in Supplementary Fig. S8(b). The solid curve was obtained by using a broadening of 0.1 eV for the δ-functions. Comparison with the experimental data shows that the dominant contribution to optical absorption is coming from the $\pi_1 \to \pi_1^*$ and $\pi_1 \to \pi_2^*$ transitions, where π_1 is located at -5.6 eV. We calculated the MCD spectra allowing only for these two transitions. The result is the black curve shown in Supplementary SFig. 9, which is in better agreement with the experimental data of Fig. 4(d).

Supplementary Fig. S7(b) shows that the spectral-weight functions of the π_1^* and π_2^* states overlap in energy by about 0.2 eV. We artificially eliminated this overlap by shifting the spectral weight of the π_2^* state by 0.2 eV higher in energy. Its result is shown as the red dashed curve in Supplementary Fig. S9. Hence, we obtain the best agreement with the experimental MCD data when π_1^* and π_2^* spectral weights have no overlap.

QMC measurements

In the DFT+QMC approach, the Coulomb interactions at the Fe($3d_\nu$) orbitals are taken into account by both the DFT and the QMC techniques. In order to prevent this double counting, an orbital-dependent double-counting term μ_ν^{DC} defined as

$$\mu_\nu^{\mathrm{DC}} = \frac{1}{2} U\, n_{d\nu}^0 + \frac{1}{2}(U' + U'') \sum_{\nu' \neq \nu} n_{d\nu'}^0 \tag{3}$$

is substracted from the bare Fe($3d_\nu$) energy levels, $\varepsilon_{d\nu} \to \varepsilon_{d\nu} - \mu_\nu^{\mathrm{DC}}$. Here, $n_{d\nu}^0$ is the electron number in the Fe($3d_\nu$) NAO's obtained by the DFT calculations.

By using QMC simulations we calculate the expectation values of various operators to study the electronic properties of the effective Anderson model for heme. In particular, we calculate the distribution of the magnetic moments in the cluster, the correlations among these moments, the magnetic susceptibilities and in addition the charge distribution throughout the cluster. The electron occupation number of the Fe($3d_\nu$) orbitals is obtained from

$$\langle n_\nu \rangle = \sum_\sigma \langle d_{\nu\sigma}^\dagger d_{\nu\sigma} \rangle, \tag{4}$$

where the expectation value of an operator A is defined by

$$\langle A \rangle = \frac{1}{Z} \mathrm{Tr}\, e^{-\beta H} A \tag{5}$$

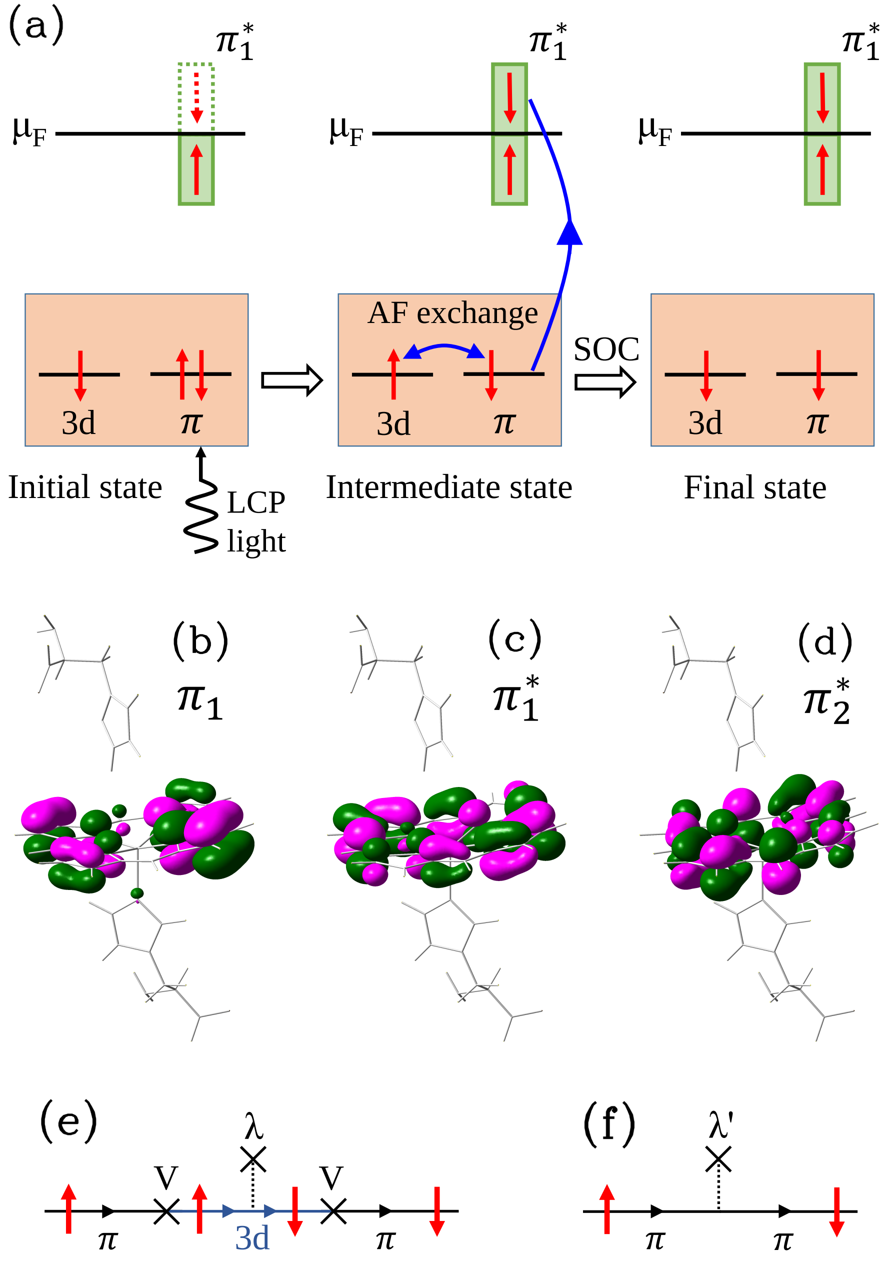

Supplementary Fig. S6 Electronic transitions for the anomalous MCD spectrum of deoxy-heme

(a) Illustration of the initial, intermediate and the final states in the $\pi \to \pi_1^*$ transition with LCP light absorption in MCD in the UV region. These panels are for an applied magnetic field pointing in the up direction, which is also along the direction of light propagation. (b)-(d) Illustration of the wavefunctions for the bonding π_1 and the antibonding π_1^* and π_2^* host states, respectively. (e) Feynman diagram illustrating a hybridization process through which an electron in a bonding π state gains an effective spin-orbit coupling. Here, an up-spin electron in the π state can become an up-spin 3d electron through hybridization. Because of the spin-orbit coupling at the Fe site, this up-spin 3d electron can now flip its spin down. Through hybridization for a second time, it then becomes a down-spin π electron. This is the lowest-order diagram to the set of processes where the π state gains an effective spin-orbit coupling. Here, V is the hybridization matrix element between the π state and the Fe(3d) orbitals, and λ is the spin-orbit coupling constant for the Fe(3d) orbitals. (f) Because of processes as shown in (e), an electron in a π state can gain an effective spin-orbit coupling λ'.

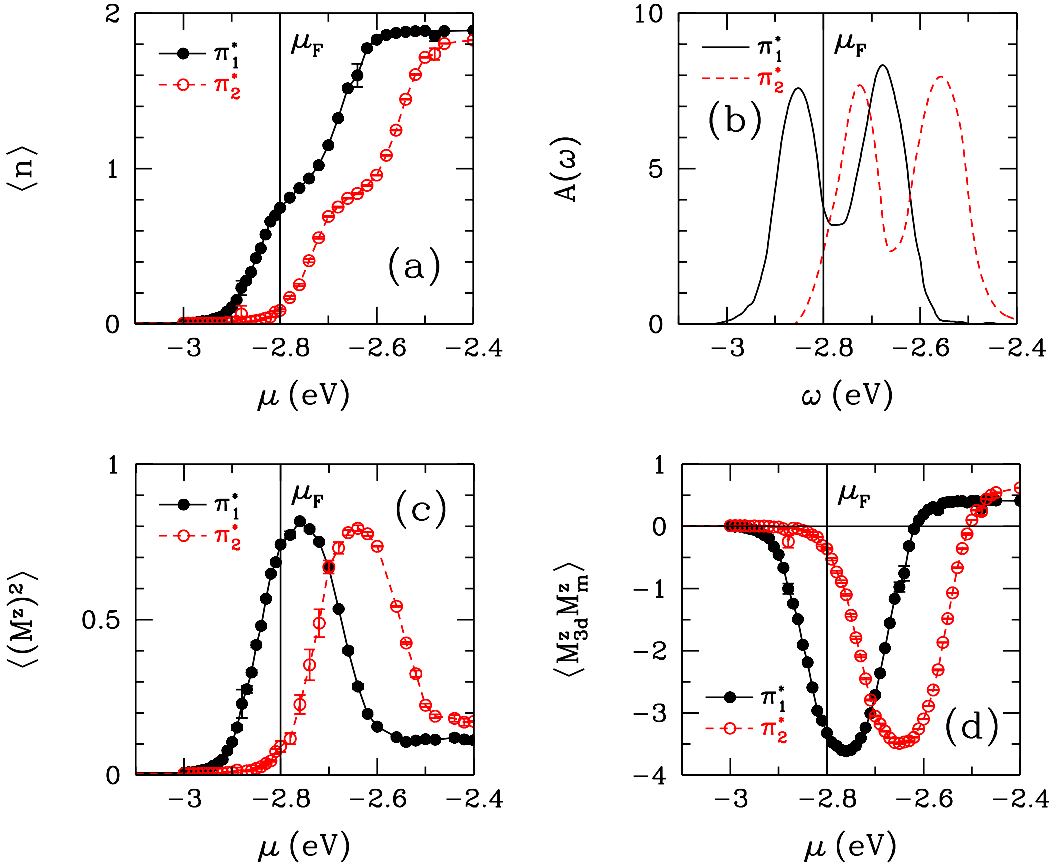

Supplementary Fig. S7 Electronic properties of the π_1^* and π_2^* host states located near the Fermi level in deoxy-heme

(a) Electron occupation $\langle n \rangle$ of the π_1^* and π_2^* host states plotted as a function of the chemical potential μ. (b) Single-particle spectral weight $A(\omega)$ versus ω for the π_1^* and π_2^* states obtained from the $\langle n \rangle$ versus μ results shown in (a). (c) Square of the magnetic moment of the π_1^* and π_2^* states versus μ. (d) Magnetization correlation function of the Fe($3d$) magnetic moment with the moments of the π_1^* and π_2^* states $\langle M^z_{3d} M^z_m \rangle$ versus μ. In these figures, the black vertical line denotes the Fermi level μ_F, and the results are presented for $T = 300$ K.

with $Z = \mathrm{Tr}\, e^{-\beta H}$ the partition function. In addition, we calculate the effective magnetic moments M_ν^{eff} of the Fe($3d_\nu$) orbitals from

$$M_\nu^{\mathrm{eff}} = \sqrt{\langle (M_\nu^z)^2 \rangle}, \tag{6}$$

where the longitudinal magnetization operator for the Fe($3d_\nu$) orbital is defined in units of μ_{B} as

$$M_\nu^z = d^\dagger_{\nu\uparrow} d_{\nu\uparrow} - d^\dagger_{\nu\downarrow} d_{\nu\downarrow}. \tag{7}$$

We calculate the total Fe($3d$) spin susceptibility from

$$\chi_{3d} = \int_0^\beta d\tau \, \langle M^z_{3d}(\tau) M^z_{3d}(0) \rangle \tag{8}$$

where the total Fe($3d$) magnetization operator is $M^z_{3d} = \sum_\nu M^z_\nu$ with the Matsubara-time evolution $M^z_{3d}(\tau) = \exp(H\tau) M^z_{3d} \exp(-H\tau)$. Similarly, the total spin susceptibility of the cluster is obtained from

$$\chi_{\mathrm{t}} = \int_0^\beta d\tau \, \langle M^z_{\mathrm{t}}(\tau) M^z_{\mathrm{t}}(0) \rangle \tag{9}$$

where the total magnetization operator of the cluster is $M^z_{\mathrm{t}} = M^z_{3d} + M^z_{\mathrm{h}}$ and the total magnetization operator of the host is $M^z_{\mathrm{h}} = \sum_m M^z_m$ with

$$M^z_m = c^\dagger_{m\uparrow} c_{m\uparrow} - c^\dagger_{m\downarrow} c_{m\downarrow} \tag{10}$$

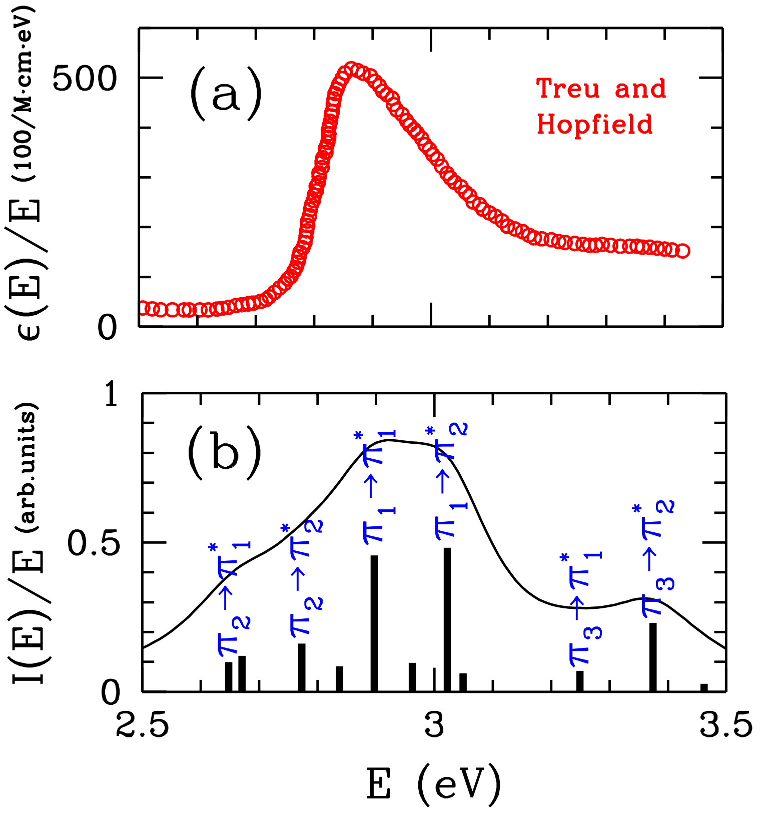

Supplementary Fig. S8 Experimental and calculated results on the optical absorption in UV region for deoxy-heme

(a) Experimental data on the frequency dependence of the optical absorption normalized by energy $\varepsilon(E)/E$ for deoxy-HbA by Treu and Hopfield[12]. (b) Calculated optical absorption normalized by energy $I(E)/E$ for deoxy-heme. The black bars denote the weights of the various $\pi \to \pi^*$ transitions. Here, we have labelled the π states which give the leading contributions as π_1, π_2 and π_3. We have also indicated which particular $\pi \to \pi^*$ transitions the bars correspond to. The black curve was obtained by artificially broadening the delta functions by 0.1 eV.

in units of $\mu_{\rm B}$. In evaluating the integrand in Eq. (10), we include the correlations among the Fe($3d_\nu$) orbitals, and the Fe($3d$)-host correlations in addition to the intra-orbital host correlations as seen in

$$\langle M_{\rm t}^z(\tau) M_{\rm t}^z(0)\rangle = \sum_{\nu,\nu'} \langle M_\nu^z(\tau) M_{\nu'}^z(0)\rangle + 2\sum_{\nu,m} \langle M_\nu^z(\tau) M_m^z(0)\rangle + \sum_m \langle M_m^z(\tau) M_m^z(0)\rangle. \tag{11}$$

However, we do not include the contribution coming from the inter-orbital host correlations

$$\sum_{m,m'\neq m} \langle M_m^z(\tau) M_{m'}^z(0)\rangle, \tag{12}$$

because its effect on $\chi_{\rm t}$ is negligible compared to the other terms. In the oxy-heme case, we estimate its contribution to $\chi_{\rm t}$ to be less than $\approx 4\,\mu_{\rm B}^2/{\rm eV}$.

Calculation of the magnetic-moment density

Here, we describe how we calculate the magnetic-moment density $M(\mathbf{r})$ for the heme clusters. The effective magnetic moment of the m'th host state is obtained from

$$M_m^{\rm eff} = \sqrt{\langle (M_m^z)^2\rangle}. \tag{13}$$

While constructing the effective Anderson Hamiltonian for the heme cluster from the Fock matrix, we have obtained the expansion of the host states in terms of the atomic orbitals,

$$c_{m\sigma} = \sum_i D_{m,i}\, \tilde{c}_{i\sigma}, \tag{14}$$

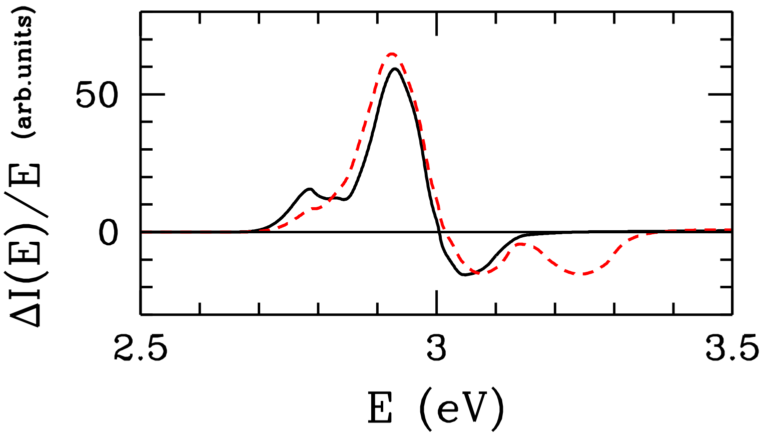

Supplementary Fig. S9 MCD spectrum due to $\pi_1 \to (\pi_1^*, \pi_2^*)$ optical transitions

Here, the black curve denotes the MCD spectrum for when only the $\pi_1 \to \pi_1^*$ and $\pi_1 \to \pi_2^*$ transitions are taken into account. The red-dashed curve denotes the same in the case where the π_2^* state is artificially shifted by 0.2 eV to higher energies so that there is no overlap with the π_1^* state. This yields the best agreement with the experimental line shape seen in Fig. 4(d).

where $\tilde{c}_{i\sigma}$ destroys an electron with spin σ in the i'th atomic orbital. Hence, the magnetization operator of the m'th host state can be written as

$$M_m^z = \sum_{i,j} D_{m,i}^* D_{m,j} (\tilde{c}_{i\uparrow}^\dagger \tilde{c}_{j\uparrow} - \tilde{c}_{i\downarrow}^\dagger \tilde{c}_{j\downarrow}). \tag{15}$$

In order to obtain a simple illustration of the magnetization density, we neglect the correlations for when $i \neq j$, and define the following approximate expression as the magnetic-moment density for the host states

$$M(\mathbf{r}) \approx \sum_{m,i} M_m^{\mathrm{eff}} \, |D_{m,i}|^2 \, \delta(\mathbf{r} - \mathbf{r}_i), \tag{16}$$

where $\mathbf{r}_i$ is the coordinate of the i'th atomic orbital. Using this expression, we show in Fig. 2 the Fe($3d$) and the host magnetic-moment density in the basis of the atomic orbitals as a bubble graph. We use the sign of the Fe($3d$)-host correlation function $\langle M_{3d}^z M_m^z \rangle$ to determine the sign of $M(\mathbf{r})$. In obtaining $M(\mathbf{r})$ from Eq. (17), we have only included the contributions from host states with $|M_m^{\mathrm{eff}}| > 0.1\,\mu_{\mathrm{B}}$, since it is difficult to obtain M_m^{eff} accurately for when $\langle (M_m^z)^2 \rangle < 0.01\,\mu_{\mathrm{B}}^2$. Hence, Fig. 2 does not show the long-range tail of the magnetic-moment density away from the Fe site. It is cut-off when $|M(\mathbf{r})| \lesssim 0.1\mu_{\mathrm{B}}$.

References

1. Reed, A. E., Curtiss, L. A. & Weinhold, F. Intermolecular interactions from a natural bond orbital, donor-acceptor viewpoint. Chem. Rev. **88**, 899 (1988).
2. Kandemir, Z. Mapping of the electronic structure of metalloproteins onto multi-orbital Anderson model using the density functional theory, Master's thesis, Izmir Institute of Technology, (2013), available at https://openaccess.iyte.edu.tr/bitstream/handle/11147/3626/10013875.pdf.
3. Kandemir, Z., Mayda, S. & Bulut, N. Electronic structure and correlations of vitamin B_{12} studied within the Haldane-Anderson impurity model. Eur. Phys. J. B **89**, 113 (2016).
4. Mayda, S., Kandemir, Z. & Bulut, N. Electronic structure of cyanocobalamin: DFT+QMC study. J. Supercond. Nov. Magn., **30**, 3301 (2017).
5. Frisch, M. J. et al.: Gaussian 09, Revision D.01. Gaussian, Inc., Wallingford, CT (2009).
6. Becke, A. D. Density-functional exchange-energy approximation with correct asymptotic behavior. Phys. Rev. A **38**, 3098 (1988).
7. Perdew, J. P. Density-functional approximation for the correlation energy of the inhomogeneous electron gas. Phys. Rev. B **33**, 8822 (1986).
8. Weber, C., O'Regan, D. D. O., Hine, N. D. M., Littlewood, P. B., Kotliar, G. & Payne, M. C. Importance of Many-Body Effects in the Kernel of Hemoglobin for Ligand Binding, Phys. Rev. Lett. **110**, 106402 (2013).

9. Chiesa, A., Carretta, S., Santini, P., Amoretti, G. & Pavarini, E. Many-Body Models for Molecular Nanomagnets, *Phys. Rev. Lett.* **110**, 157204 (2013).

10. Birukou, I., Schweers, R. L. & Olson, J. S. Distal histidine stabilizes bound O_2 and acts as a gate for ligand entry in both subunits of adult human hemoglobin. J. Bio. Chem. **285**, 8840 (2010).

11. Chen, J., Millis, A. J. & Marianetti, C. A. Density functional plus dynamical mean-field theory of the spin-crossover molecule $Fe(phen)_2(NCS)_2$, *Phys. Rev. B* **91**, 241111(R) (2015).

12. Treu, J. I. & Hopfield, J. J. Magnetic circular dichroism in hemoglobin. *J. Chem. Phys.* **63**, 613 (1975).
